# Functional network structure supports resilience to memory deficits in cognitively normal older adults with amyloid-β pathology

**DOI:** 10.1038/s41598-023-40092-x

**Published:** 2023-08-25

**Authors:** Jenna N. Adams, Miranda G. Chappel-Farley, Jessica L. Yaros, Lisa Taylor, Alyssa L. Harris, Abanoub Mikhail, Liv McMillan, David B. Keator, Michael A. Yassa

**Affiliations:** 1 Department of Neurobiology and Behavior, University of California, Irvine, 1400 Biological Sciences 3, Irvine, CA 92697-3800 USA; 2 Center for the Neurobiology of Learning and Memory, University of California, Irvine, Irvine, CA 92697 USA; 3 Department of Psychiatry and Human Behavior, University of California, Irvine, 1418 Biological Sciences 3, Irvine, CA 92697-3800 USA

**Keywords:** Cognitive ageing, Alzheimer's disease, Long-term memory

## Abstract

Older adults may harbor large amounts of amyloid-β (Aβ) pathology, yet still perform at age-normal levels on memory assessments. We tested whether functional brain networks confer resilience or compensatory mechanisms to support memory in the face of Aβ pathology. Sixty-five cognitively normal older adults received high-resolution resting state fMRI to assess functional networks, 18F-florbetapir-PET to measure Aβ, and a memory assessment. We characterized functional networks with graph metrics of local efficiency (information transfer), modularity (specialization of functional modules), and small worldness (balance of integration and segregation). There was no difference in functional network measures between older adults with high Aβ (Aβ+) compared to those with no/low Aβ (Aβ−). However, in Aβ+ older adults, increased local efficiency, modularity, and small worldness were associated with better memory performance, while this relationship did not occur Aβ− older adults. Further, the association between increased local efficiency and better memory performance in Aβ+ older adults was localized to local efficiency of the default mode network and hippocampus, regions vulnerable to Aβ and involved in memory processing. Our results suggest functional networks with modular and efficient structures are associated with resilience to Aβ pathology, providing a functional target for intervention.

## Introduction

The pathologies characteristic of Alzheimer’s disease (AD), namely aggregates of amyloid-β (Aβ) as amyloid plaques and hyperphosphorylated tau proteins as neurofibrillary tangles, begin to develop decades before the onset of frank clinical symptoms^1,2^. To this effect, a significant proportion (20–30%) of older adults who are cognitively normal for their age group harbor elevated levels of Aβ pathology^3^. Understanding how some older adults with high levels of AD pathology remain cognitively healthy is a critical research question.

This preservation of cognition may occur through mechanisms such as resilience^4,5^ or compensation^4^. Cognitive reserve, a type of resilience mechanism defined as the anatomical and neurophysiological resources that help allow the brain to withstand insult^4,5^, may be one such method of resilience to pathology. Cognitive reserve may be developed throughout the lifespan by interactions between genetic^6^ and environmental factors^7^, and may exist prior to age- or disease-related neural insult^4,5^. In contrast, compensation reflects the ability of the brain to dynamically recruit additional neural resources to meet current cognitive demands, counteracting age- or disease-related cognitive decline, and may be more specific to the cognitive demand at hand^4^.

Functional brain networks, which reflect coordinated neural activity that can be measured with functional MRI^8^, are a compelling mechanism which may support resilience or compensation to pathology. Communication among brain regions can dynamically reconfigure in response to task demands^9^, and functional networks measured at rest have been shown to change across the lifespan^10^. Communication among elements of a network can be characterized using *graph theory*^11,12^. Graph theory models the complex interactions of the brain as a “graph”, with brain regions represented as “nodes” and the functional connections between pairs of regions represented as “edges”. Focusing on the strongest edges, the network can be reduced and subsequently analyzed to quantify meaningful topological characteristics, such as measures representing efficiency of information transfer (i.e. *local efficiency*^13,14^), specialization of functional modules (i.e. *modularity*^15^), and balance of integration and segregation (i.e. *small worldness*^16,17^). Prior studies have found that these graph metrics are related to better cognition in healthy individuals^18–20^.

Graph theory has also been applied to examine how functional brain networks change with aging and disease. Overall, aging is associated with reductions in graph metrics such as local efficiency, modularity, segregation, and small worldness^21–28^, and these differences are exaggerated in patients with AD^21,29,30^. However, few studies have examined whether graph measures of functional networks relate to preserved cognition in preclinical AD. A recent study by Ewers and colleagues^31^ showed that increased system segregation of major functional networks was associated with better-than-expected cognition at equivalent levels of pathology in patients with AD, providing compelling evidence that modularization of functional networks may be a mechanism of resilience. However, whether this effect extends to cognitively normal older adults is still unknown.

The goal of our study was to test whether the structure of functional networks, characterized with graph theory, provide an early potential mechanism for resilience or compensation, enabling older adults to remain cognitively normal in the face of emerging AD pathology. We specifically focused on memory performance, as memory is the first domain to begin to exhibit decline in preclinical AD. We investigated a sample of cognitively normal older adults with high-resolution resting state fMRI to measure graph metrics of functional networks (i.e. local efficiency, modularity, and small worldness), and 18F-florbetapir PET to measure Aβ pathology.

We hypothesized that if functional networks provided a *compensatory* response to pathology, we would observe differences in network measures in Aβ+ compared to Aβ- older adults, which would also be related to better memory performance. Higher values of network measures in the Aβ+ group would suggest that these participants have an alteration of functional resources at rest compared to their Aβ− counterparts, which may differentially impact memory performance when these resources are later recruited, suggesting compensation. In contrast, if functional networks provide a *resilience* or *cognitive reserve* mechanism to emerging pathology, we would predict no Aβ-related differences in network measures, as this network structure would be pre-existing and not change in response to pathology. However, increased network measures would be related to better memory performance in Aβ+ older adults, providing a mechanism to overcome Aβ pathology.

## Results

### Demographics

Sixty-five cognitively normal older adults from the Biomarker Exploration in Cognition, Aging and Neurodegeneration (BEACoN) study at UC Irvine who received both resting state fMRI and 18F-florbetapir (FBP) PET were included in the present analysis. Participant demographics are presented in Table 1. Using a validated global FBP SUVR threshold to determine Aβ status^32^, 21 participants (32%) were identified as Aβ+ . There were significantly more female participants in the Aβ+ group, and a trend for older age (see Table 1 for group comparisons). As such, age and sex, as well as education, were included as covariates in all models.

**Table 1:** Demographics of the sample.

	Total Sample	Aβ-	Aβ+	Aβ- vs. Aβ+	
	M ± SD or N (%)	M ± SD or N (%)	M ± SD or N (%)	*t* or *X*^2^	*p*
Age	72.3 ± 6.2	71.3 ± 5.7	74.4 ± 6.8	-1.92	0.06
Sex (Female)	43 (66.2%)	18 (40.9%)	17 (81.0%)	8.91	0.003
Education (Years)	16.2 ± 2.3	16.3 ± 2.5	16.1 ± 2.0	0.36	0.72
Race (White)	52 (80%)	33 (75%)	19 (90.5%)	2.13	0.15
Ethnicity (Non-Hispanic)	63 (96.9%)	43 (97.7%)	20 (95.2%)	0.30	0.59
MMSE	28.3 ± 1.4	28.3 ± 1.4	28.5 ± 1.4	-0.55	0.59
RAVLT Immediate	12.7 ± 2.0	12.6 ± 2.2	13.0 ± 1.7	-0.67	0.51
FBP Mean SUVR	1.10 ± 0.18	1.00 ± 0.07	1.32 ± 0.15	-12.31	< 0.001
Aβ+	21 (32.3%)	–	–	–	–

Aβ, amyloid-β,; MMSE, Mini Mental State Exam; RAVLT, Rey Auditory Verbal Learning Task; FBP, 18F-florbetapir-PET; SUVR, standardized uptake value ratio; M, mean; SD, standard deviation; N, number.

There was no difference between Aβ+ and Aβ− older adults in memory performance (Table 1), assessed with word-list recall (see *Methods*). This indicates that even with high levels of Aβ pathology, Aβ+ participants are on average able to retain equivalent memory performance to older adults without Aβ pathology.

### Characterization of functional networks with graph analysis

**Figure 1:**
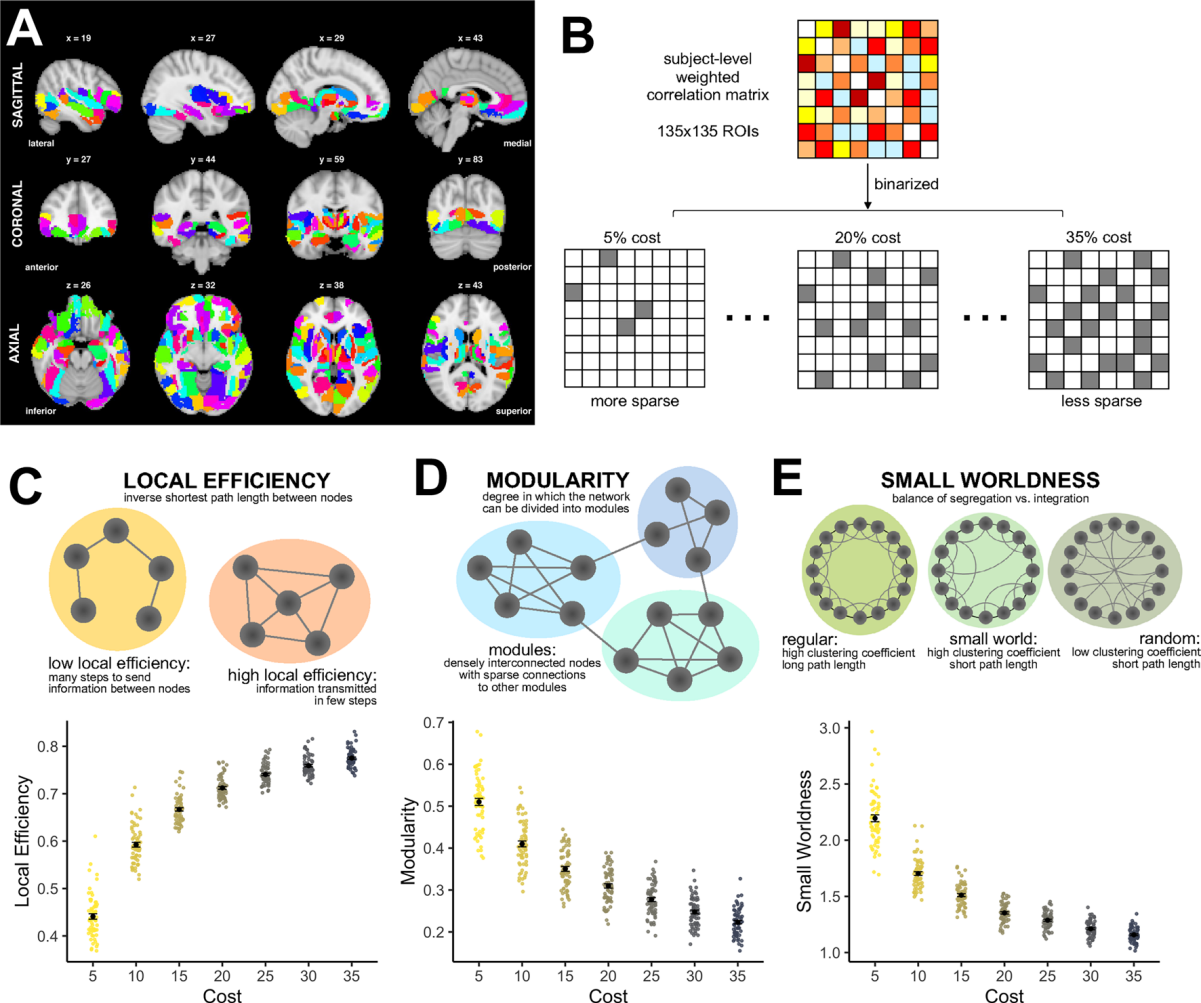
Overview of graph theoretical methods applied to resting state functional MRI. **(A)** 135 regions of interest (ROIs) from the Brainnetome Atlas were included within the partial field of view in all participants and used for graph theory analysis. **(B)** BOLD time series were extracted from each ROI and correlated across ROIs to obtain an 135 × 135 connectivity matrix for each participant. Correlation matrices were then binarized at 7 cost levels, ranging from 5% cost (keeping the 5% strongest connections, most sparse) to 35% cost (keeping the 35% strongest connections, less sparse) using 5% step sizes. The graph metrics of local efficiency **(C)**, modularity **(D)**, and small worldness **(E)** were calculated at each cost level.

**Figure 2:**
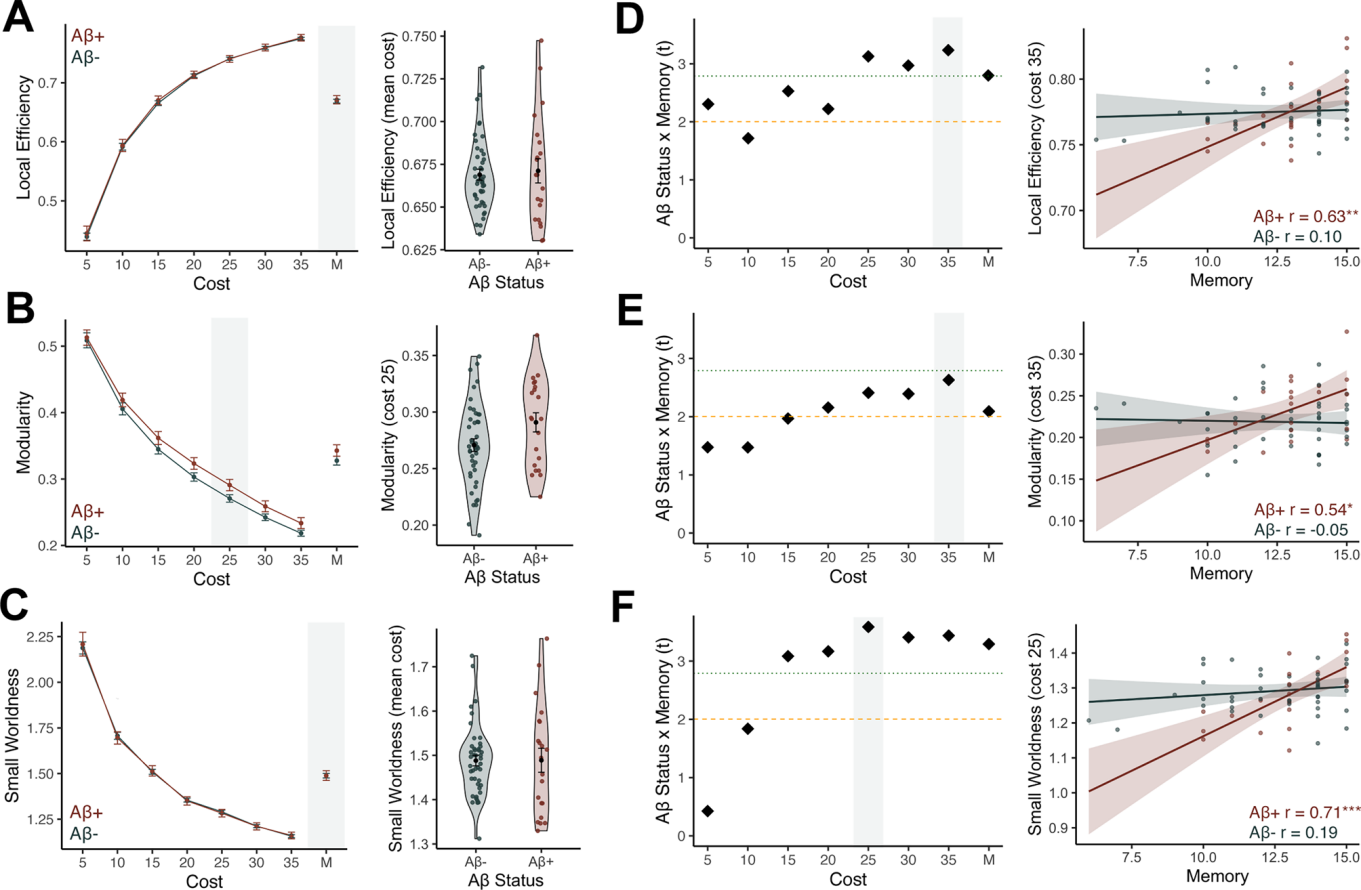
Graph metrics of functional brain networks are related to memory performance in Aβ+ older adults. **(A–C)** We tested for group differences in graph metrics between Aβ+ and Aβ- older adults at each cost level using repeated measures ANCOVA analysis, controlling for age, sex, and education. There was no significant main effect of Aβ group or Aβ group by cost interaction, indicating no differences in graph metrics between the groups. Left side plots demonstrate the group mean and standard error at each cost, with Aβ+ group in red, and the Aβ- group in navy. The right hand plot shows a closer look at this comparison at one cost, indicated by the shading, at either the strongest cost or at the mean of costs. **(D–F)** We tested for an interaction between Aβ status and memory performance at each cost to determine if the relationship between memory and graph metrics differ by Aβ status. This was performed using repeated measures ANCOVAs at each cost, with the Aβ status by memory interaction being our outcome of interest. We performed follow-up linear regressions at each cost, with the t-statistic (diamond shape) of the interaction between Aβ status and memory being plotted on the left side. The t-statistic crossed the *p* < 0.05 significance threshold (dashed yellow line) and p-FDR significance threshold (< 0.0071, dotted green line) for the majority of costs. The gray shaded cost levels are plotted on the right side panel, showing the scatter plot of the interaction. Higher local efficiency **(D),** modularity **(E)**, and small worldness **(F)** was significantly associated with better memory performance in the Aβ group (red) but not the Aβ− group (navy). *p < 0.05; **p < 0.01; ***p < 0.001.

We focused on three established graph theoretical measures of functional networks (see Fig. 1C–E): (1) *local efficiency*, a measure of information transfer, calculated as the inverse shortest path length computed on the neighborhood of the node^13,14^, with high local efficiency enabling robustness to a removed node^24^ (Fig. 1C); (2) *modularity*, the ability of a network to be reduced into specialized modules, which exhibit strong connectivity within the module and sparse connectivity between modules^15^ (Fig. 1D); and (3) *small worldness*, a measure indicating balance between segregation and integration within the network, which is characterized by high clustering coefficient and short path lengths between functionally related regions^16,17^ (Fig. 1E).

To derive these measures, BOLD timeseries from resting state fMRI were extracted from 135 regions of interest (ROIs) from the Brainnetome Atlas included within spatial coverage of the scan (Fig. 1A; see Supplementary Table 1 for detailed ROI information). Functional imaging was focused across the temporal lobe, including critical parts of the parietal, occipital, and frontal lobe, to enable higher spatial resolution to better obtain signal from distinctive ROIs however, we acknowledge that this partial coverage may influence graph metrics. ROI-to-ROI correlation matrices were generated for each participant, and binarized across a range of costs to vary the sparsity level of the network (Fig. 1B). Network sparsity was calculated from 5% cost (top 5% strongest connections in the network, highly sparse) to 35% cost (less sparse) in 5% step sizes (7 total costs). This cost range was selected a priori because it has been proposed to ideally characterize small-world characteristics of brain networks^19,24,33^. Graph metrics were then calculated at each cost with the Brain Connectivity Toolbox^13^. Figure 1C–E visualizes the distribution of graph values across costs. Analyses relating graph metrics to Aβ status and memory performance were performed across all costs to further substantiate that results were not driven by arbitrary cost selection, with cost included as a repeated subjects measure, to test for consistency in the effects across cost.

### Network structure does not differ between Aβ groups

Our first aim was to test whether local efficiency, modularity, and small worldness differed by Aβ status in cognitively normal older adults. To test this, we conducted a repeated measures ANCOVA analyses for each graph metric, including the value at each cost as the repeated measure (7 costs), Aβ status as the between subjects factor, and age, sex, and education as covariates. Our outcomes of interest were a main effect of Aβ status and an Aβ status by cost interaction, which would indicate consistent versus specific effects of Aβ on each graph metric. There was no significant main effect of Aβ status or Aβ status by cost interaction for local efficiency (main effect: F(1) = 0.001, *p = *0.98); interaction: F(1.86) = 0.12, *p = *0.87; Fig. 2A), modularity (main effect: F(1) = 1.20, *p = *0.28); interaction: F(1.42) = 0.97, *p = *0.36; Fig. 2B), or small worldness (main effect: F(1) = 0.11, *p = *0.74); interaction: F(1.49) = 0.34, *p = *0.65; Fig. 2C), indicating that these graph metrics did not vary by Aβ status. Figure 2 demonstrates a visualization of the group comparisons at each cost, as well as a closer examination of the distributions at specific costs.

### Memory performance is associated with network structure in Aβ+ older adults

We next investigated whether memory performance, defined by free recall on a word list learning task (see *Methods*), was associated with local efficiency, modularity, and small worldness regardless of pathological load. To test this, we constructed similar repeated measures ANCOVAs, but with memory performance as a continuous predictor, again focusing on a main effect of memory or a memory by cost interaction. Overall, better memory performance was associated with greater small worldness (main effect: F(1) = 6.52, *p = *0.01), which did not significantly vary by cost (memory by cost interaction: F(1.49) = 1.11, *p = *0.32). There was also a trend for memory performance being associated with overall higher local efficiency (main effect: F(1) = 3.93, *p = *0.052; memory by cost interaction: F(1.86) = 0.38, *p = *0.67). There was no effect of modularity on memory (main effect: F(1) = 0.002, *p = *0.97; memory by cost interaction: F(1) = 0.70, *p = *0.41).

Our primary goal was to determine whether the relationship between memory performance and network structure was dependent on Aβ status. To test this, we included both memory and Aβ status in the repeated measures ANCOVA models, and focused on the interaction between memory and Aβ status as our outcome of interest to probe the impact of amyloid load as a function of memory performance on each graph metric. We found strong memory by Aβ status interactions in predicting local efficiency (F(1) = 7.84, *p = *0.007), modularity (F(1) = 4.37, *p = *0.04), and small worldness (F(1) = 10.83, *p = *0.002), indicating that the relationship between memory and graph metrics was highly dependent on Aβ status. The three-way interaction between memory, Aβ status, and cost was not significant, indicating that this effect was consistent across cost levels (local efficiency: F(1.86) = 1.00, *p = *0.37; modularity: F(1.39) = 0.11, *p = *0.83; small worldness: F(1.51) = 2.28, *p = *0.12).

We closely examined the memory by Aβ status interaction for each graph metric across costs when our interaction of interest reached significance, depicted in Fig. 2D–F. For each cost, we conducted follow-up linear regression models including the Aβ status by memory interaction and covariates including age, sex, and education. The interaction between Aβ status and memory was significant across the majority of costs for each graph metric (ps < 0.05; Fig. 2D–F), replicating the results of the ANCOVA. Then, to determine which Aβ group was driving the interactions, we tested the correlations between memory and each graph measure within each Aβ group separately, controlling for age, sex, and education. In the Aβ+ group, better memory performance was associated with higher local efficiency (e.g., cost 35: r = 0.63; *p = *0.005; Fig. 2D), higher modularity (e.g., cost 25: r = 0.54, *p = *0.02; Fig. 2E), and greater small worldness (e.g., cost 25: r = 0.71, *p* < 0.001; Fig. 2F). In the Aβ- group, there were no significant relationships between memory and local efficiency (e.g., cost 35: r = 0.10, *p = *0.52; Fig. 2D), modularity (e.g., cost 35: r = − 0.05, *p = *0.75; Fig. 2E), or small worldness (e.g., cost 25: r = 0.19, *p = *0.25; Fig. 2F) at any cost (all ps > 0.05). Additionally, comparing the strength of correlations between Aβ+ and Aβ− groups with Fisher’s *r* to *z* transformations indicated that the association between memory and graph metrics were significantly stronger in the Aβ+ group compared to the Aβ− group (ps < 0.05 for all costs). These results suggest that the association between better memory performance and graph metrics of functional networks occurs specifically within participants with high levels of Aβ burden.

### Default mode network local efficiency is related to memory performance in Aβ+ older adults

Because local efficiency is calculated on the level of the node (see ***Methods***), we next asked whether the local efficiency of specific subnetworks and regions varied by Aβ status and had Aβ-specific associations with memory as a secondary goal. We chose to investigate local efficiency of the default mode network (DMN), as this network overlaps spatially with regions that first accumulate Aβ pathology^34^ and contributes to memory processing^35^.

**Figure 3:**
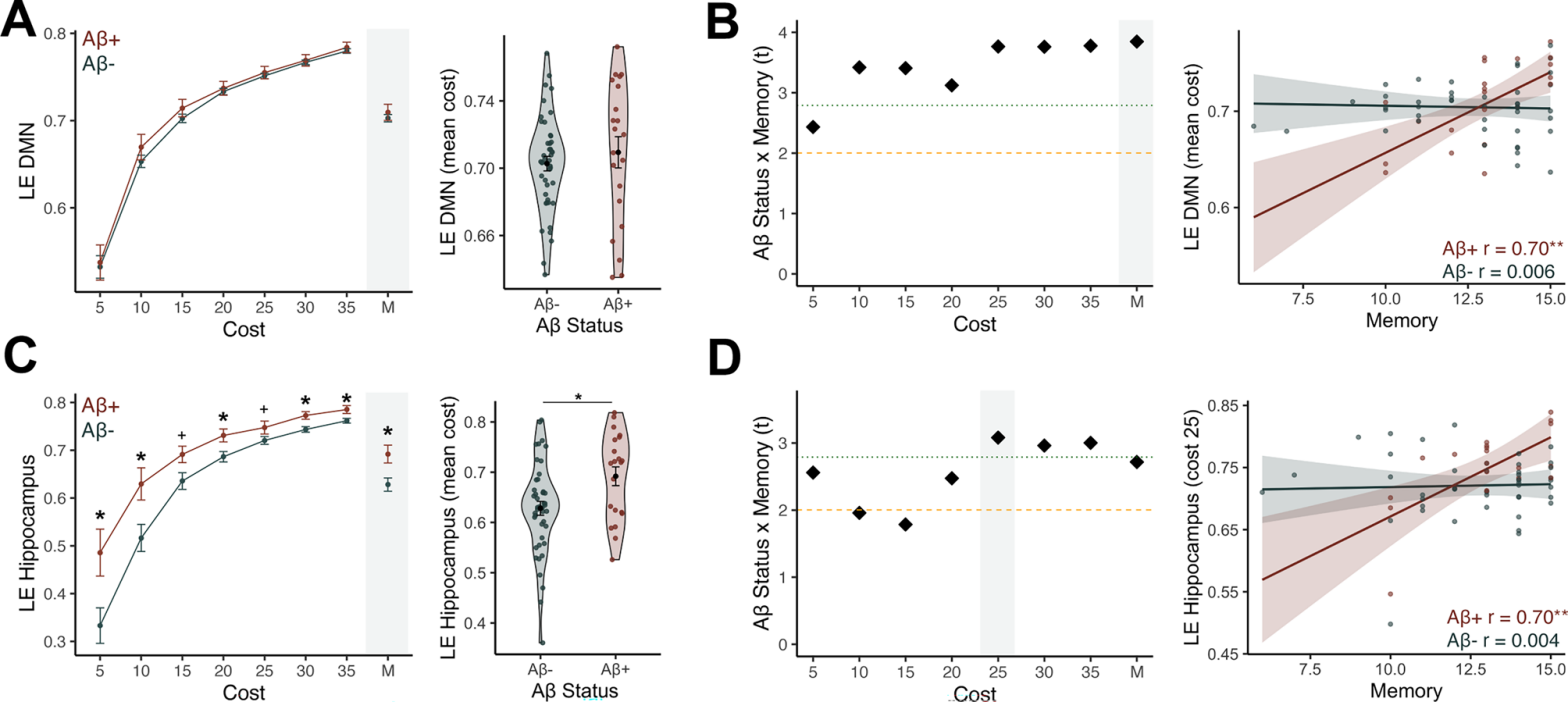
Local efficiency of the default mode network (DMN) and hippocampus are related to memory performance in Aβ+ older adults. **(A)** There was no significant difference in local efficiency in the DMN between the Aβ+ (red) and Aβ− (navy) groups. Left side plots demonstrate the group mean and standard error at each cost, and the right hand plot shows a closer look at this comparison at the mean cost, indicated by gray shading. **(C)** The Aβ+ group had significantly higher hippocampal local efficiency compared to the Aβ− group at 5 out of 7 costs. This effect is plotted for the mean of costs on the right side panel, indicated by the gray shading. **(B/D)** We tested for an interaction between Aβ status and memory performance to DMN local efficiency **(B)** and hippocampal local efficiency **(D)** at each cost to determine if the relationship between memory and local efficiency differs by Aβ status. We performed follow-up linear regressions at each cost, with the t-statistic (diamond shape) of the interaction between Aβ status and memory being plotted on the left side. The t-statistic crossed the *p* < 0.05 significance threshold (dashed yellow line) and p-FDR significance threshold (< 0.0071, dotted green line) for the majority of costs. The gray shaded cost levels are plotted on the right side panel, showing the scatter plot of the interaction. Higher DMN local efficiency **(B),** and hippocampal local efficiency **(D)** were significantly associated with better memory performance in the Aβ group (red) but not the Aβ- group (navy). Control analyses using local efficiency of the visual network in place of the DMN and the superior temporal gyrus in place of the hippocampus did not produce significant group differences or Aβ x memory interactions, highlighting the specificity of these findings to local efficiency of the DMN and hippocampus. *p < 0.05; **p < 0.01; ***p < 0.001.

We first investigated Aβ-related changes to DMN local efficiency, constructing similar repeated measures ANCOVA as in the whole network models but including DMN local efficiency at each cost as the repeated measures factor. Consistent with the whole brain local efficiency analysis, there was no significant main effect of Aβ status (F(1) = 0.05, *p = *0.83) or Aβ status by cost interaction (F(1.48) = 0.19, *p = *0.76), depicted in Fig. 3A.

We next tested main effects of memory and memory by Aβ status interactions in predicting DMN local efficiency. While there was no significant main effect of memory (F(1) = 2.37, *p = *0.13) or memory by cost interaction (F(1.50) = 1.70, *p = *0.20), we again found a strong memory by Aβ status interaction (F(1) = 14.79, *p* < 0.001) that did not significantly vary by cost (F(1.5) = 2.60, *p = *0.10), indicating that the Aβ groups differed in their relationship between memory and local efficiency (Fig. 3B). Testing this interaction at each cost provided consistent results with the whole brain local efficiency measure—Aβ+ older adults had a strong relationship between better memory performance and higher DMN local efficiency (e.g., mean of costs: r = 0.70; *p = *0.001; Fig. 3B) while the Aβ- group did not demonstrate a relationship (e.g., mean of costs: r = 0.006; *p = *0.97).

We next tested the specificity of the association between memory and local efficiency within the DMN to determine if this was a particular effect related to the DMN, or just captured a common theme of local efficiency found in any network. We identified the visual network as an appropriate control for the DMN—this network was sampled at a similar rate to the DMN (61.76% vs. 61.11% of ROIs included, respectively), yet is not a network particularly prone to the development of Aβ pathology or as involved memory processing. Repeating the above analyses using local efficiency of the visual network resulted in non-significant associations with Aβ status, main effects of memory, or memory by Aβ status interactions (all ps > 0.10, see Supplementary Table 2 for all statistics). This control analysis indicates that local efficiency specifically within the DMN contributes to memory processing in Aβ+ participants, rather than this being a brain-wide phenomenon.

### Hippocampal local efficiency is higher and supports memory performance in Aβ+ older adults

We next examined local efficiency within the hippocampus because this region is critical to memory performance and is hyperactive in the presence of Aβ ^36^. We first tested for group differences in hippocampal local efficiency as a function of Aβ status. Using repeated measures ANCOVA models predicting to hippocampal local efficiency across costs as the repeated measure, we found a significant main effect of Aβ status (F(1) = 6.08, *p = *0.017) as well as a significant Aβ status by cost interaction (F(1.69) = 4.79, *p = *0.014). We examined these effects further by conducting planned follow-up pairwise comparisons (independent t-tests) between Aβ+ and Aβ- groups on hippocampal local efficiency at each cost level. The Aβ+ demonstrated significantly higher hippocampal local efficiency at 5 of 7 costs (Fig. 3C), with the strongest difference occurring at cost 30 (t(63) = 2.56, *p = *0.01). This group difference indicates that Aβ+ participants had a specific increase in local efficiency in the hippocampus that was not observed at the whole-brain or DMN-network level.

We then tested both main effects of memory and memory by Aβ status interactions in predicting hippocampal local efficiency. Again, we found no main effect of memory (F(1) = 0.13, *p = *0.72) or memory by cost interaction (F(1.62) = 0.14, *p = *0.83) in predicting hippocampal local efficiency. However, the interaction between memory and Aβ status (F(1) = 7.38, *p = *0.009) and the three-way interaction of memory, Aβ, and cost (F(1.73) = 3.58, *p = *0.04) were significant, indicating the Aβ groups differed in their relationship between memory and hippocampal local efficiency (Fig. 3D). Exploring this interaction further at each cost level indicated that this interaction was similarly driven by a strong correlation between better memory performance and higher hippocampal local efficiency in the Aβ+ group (e.g., cost 25: r = 0.70; *p = *0.001; Fig. 3D), but no significant association in the Aβ− group (e.g., cost 25: r = 0.004; *p = *0.98).

For a parallel control analysis, we examined local efficiency in the superior temporal gyrus, a lateral temporal lobe region that is not a particular site of Aβ-related hyperactivity. There was no significant effect of Aβ status or an Aβ status by memory interaction (ps > 0.69; see Supplementary Table 2 for full statistics), however, we did observe a main effect of memory on superior temporal gyrus local efficiency (F(1) = 6.14, *p = *0.014), suggesting a general role for the superior temporal gyrus in memory processing that was not dependent on Aβ status.

### Hippocampal volume is not related to network structure

Finally, we investigated whether the relationship between Aβ status, memory, and network structure was related to factors further along the cascade of decline such as neurodegeneration. We measured hippocampal volume, corrected by total intracranial volume, as a proxy of neurodegenerative processes. Hippocampal volume was significantly reduced (t(62) = 3.55, *p* < 0.001) and related to memory (r = -0.32, *p = *0.047) in Aβ+ older adults, though was not significantly related to memory performance within the full sample (r = -0.11, *p = *0.41).

We conducted similar repeated measures ANCOVA analyses predicting to each graph metric across costs, including hippocampal volume as our variable of interest, and age, sex, and education as covariates. There was no significant main effect of hippocampal volume (ps > 0.29) or hippocampal volume by cost interaction (ps > 0.63) for any of the graph metrics (see Supplementary Table 3 for detailed statistics), indicating that hippocampal volume was not closely related to whole brain functional network structure.

We next included the interaction between hippocampal volume and memory performance in these models to test whether participants with lower hippocampal volume may have associations between memory and network structure, to parallel our findings of an Aβ status by memory interaction. Again, there were no memory by hippocampal volume interactions (ps > 0.29) or three-way interactions with cost (ps > 0.55) in predicting any graph metric (see Supplementary Table 3 for detailed statistics). These null results indicate that the relationship between these metrics of functional network structure and memory are specifically related to Aβ pathology in the cognitively normal stage.

We then examined whether local efficiency in the hippocampus was related to hippocampal volume. We did not observe a significant main effect of hippocampal volume (*p = *0.93) or memory by hippocampal volume interaction (*p = *0.89) in predicting hippocampal local efficiency across costs (see Supplementary Table 3 for detailed statistics), suggesting the changes to hippocampal local efficiency are independent of volume within the hippocampus.

## Discussion

We demonstrate that the structure of functional brain networks, characterized with graph theoretical metrics, support episodic memory in cognitively normal older adults with high levels of Aβ pathology. Better episodic memory performance in Aβ+ participants was associated with higher local efficiency, representing greater efficiency of information transfer through the network, higher modularity, representing a greater degree of specialized functional subdivisions within the network, and higher small worldness, representing an ideal balance between segregation and integration. These local efficiency effects were further specific to the DMN and the hippocampus, which are highly involved in memory and known to be impacted by Aβ pathology, compared to control regions. We found no relationship between episodic memory and graph metrics in cognitively normal older adults without Aβ pathology, or between graph metrics and hippocampal volume. Our results suggest that a modular and efficient functional network structure may provide resilience to early accumulation of Aβ pathology to help maintain normal memory function.

Because we did not find Aβ-related group differences in graph metrics at the full-network level, but did observe strong associations between higher graph metrics and better memory performance specifically in Aβ+ older adults, our pattern of results better support a resilience or reserve mechanism for functional brain networks than a compensatory mechanism^4,5^. In combination with previous literature, our findings may suggest that older adults who happen to have higher values of these graph metrics, perhaps due to lifetime factors such as education or socioeconomic status^5,7,37^ which have previously been shown to provide cognitive reserve, may fare better in the face of emerging Aβ pathology.

Our finding of higher modularity being associated with better memory in Aβ+ older adults extend findings from a recent study by Ewers and colleagues demonstrating that higher system segregation, a measure closely related to modularity, is related to better cognition in patients with autosomal dominant and sporadic AD, which was interpreted to reflect resilience to AD pathology^31^. The current work suggests that this effect also occurs in older adults before the onset of overt clinical impairment and may provide a mechanism to preserve normal levels of cognitive ability in the presence of AD pathology. A previous study in cognitively normal older adults found that lower segregation between anterior temporal and posterior medial networks at baseline was associated with worse episodic memory over time^23^. While this association was not dependent on pathological load, it also suggests that higher segregation of networks, similar to modularity, may have a beneficial effect on memory in cognitively normal populations. Further, our finding that higher modularity is associated with better memory is also consistent with research demonstrating that higher baseline modularity predicts greater gains in cognitive training during interventional trials^38,39^, suggesting that modularity could be a compelling biomarker for intervention success in older adults reflecting cognitive plasticity.

In addition to modularity, we also show a novel role for small worldness in resilience to Aβ pathology. Small worldness has previously been found to decrease with age^26^, cognitive impairment^21^, and in AD patients^29^. We extend these findings by demonstrating that higher small worldness, indicating balance between segregation and integration, also supports memory performance within our full sample of cognitively normal older adults. Further, while positive associations with small worldness occurred in both Aβ+ and Aβ− groups, this effect was significantly stronger in Aβ+ participants, suggesting small worldness provides a mechanism for resilience in addition to general benefits of memory performance.

Finally, we demonstrate that higher local efficiency, both at the network level and within memory-specific subnetworks (i.e. DMN) and regions (i.e. hippocampus), supports memory performance in Aβ+ older adults. Local efficiency has previously been shown to decrease with aging^24,27^. Higher local efficiency may provide more fault-tolerance to disruptions within a network^24^, as the additional redundant connections between nodes may allow for preserved communication in the face of a dysfunctional node. In the case of aging and preclinical AD, this redundancy may be an important mechanism allowing for preserved communication even when some nodes are impacted by pathology and may not be able to efficiently process or send information themselves. This fault tolerance may be particularly critical within the DMN. The DMN is known to be especially vulnerable to the development of Aβ^34^, and previous studies have observed a breakdown of normal DMN connectivity patterns^40–44^ and also decreased graph metrics such as local efficiency^27^ and clustering coefficient^21^, a measure related to local efficiency, within the DMN. Within the hippocampus, we found higher local efficiency in Aβ+ compared to Aβ- older adults, which was associated with better memory performance in Aβ+ , indicating a possible local compensatory effect. However, as Aβ is known to promote hyperexcitability and hyperconnectivity of networks and circuits^45–47^, including the hippocampus^36,48^, this higher hippocampal local efficiency could also be a response to pathology.

While increases in graph metrics representing stronger connectivity (e.g. higher local efficiency) may be beneficial in the early stages of AD, these effects may also ultimately contribute to the spread of pathology. Greater connectivity strength and greater redundancy of connections may facilitate the spread of tau pathology, which has been shown to spread in accordance with patterns of strong functional and structural connectivity^49–51^. Compensatory increases in network measures, such as our finding of higher hippocampal local efficiency, may ultimately result in more pathological spread and progression towards AD. For example, globally connected hub regions have been shown to be facilitate the spread of tau pathology, increasing progression towards AD^52^. As the hippocampus is an early site of tau deposition^1^, and is known to be hyperexcitable in the presence of AD pathology^47^, compensatory increases in hippocampal local efficiency may promote tau seeding to downstream regions connected to hippocampus^53,54^. Using longitudinal data to test whether our observed group differences in hippocampal local efficiency is dynamically increased at a certain stage of pathological development, or is elevated across the lifespan in these subjects, is an important open question to explore.

We did not find a relationship between decreased hippocampal volume, graph metrics, and memory, suggesting that our findings may represent an early effect of Aβ accumulation prior to the detrimental effects of hippocampal atrophy. Functional activation has previously been shown to modulate the relationship between hippocampal volume and memory in a sample spanning cognitively normal and MCI patients, suggesting higher activation provided resilience to low hippocampal volume^55^. It is possible that network effects relating to hippocampal volume may emerge as participants begin to demonstrate overt memory deficits, rather than when they are still normal for their age group. While we found no relationships with hippocampal volume, we did find strong relationships between memory and hippocampal local efficiency, which substantiates previous findings suggesting changes to hippocampal function precede hippocampal neurodegeneration in the cascade leading to AD.

Limitations of the current study include the partial coverage of the high-resolution scan, which limited our ability to sample the entire brain. However, the coverage was consistent across all participants and included the full extent of the temporal lobe, which is a critical region for memory processing. Therefore, this spatial coverage was appropriate for our investigation into memory function. Associations between Aβ and resting state networks such as the DMN may have been stronger if the full extent of regions such as the parietal lobe were sampled. However, subnetwork analyses were a secondary goal to provide greater mechanistic insight and should be considered exploratory. Future studies with whole brain coverage should further replicate and expand on the current findings, as the specific regions contained within our scan field of view may have influenced our results. Additionally, while we defined graph metrics based on resting state networks, which provides a task-invariant assessment of functional networks, research investigating how graph metrics computed during task-based fMRI are related to performance will further speak to the potential compensatory reconfiguration of networks to support specific task demands.

Next, our sample of Aβ+ older adults was relatively small, limiting our power. However, Aβ+ participants represented 32% off our sample, consistent with incidence reported previously^3^, and the strength of associations found within this sample speak to the robustness of the findings. Further, our use of free recall during word list learning as our primary memory outcome measure may not fully capture non-dominant hemisphere function, and may be influenced by executive function strategies supported by the frontal lobe. Future work using multiple memory measures summarized together as a composite score may reflect memory processes invariant to the specific memory assessment chosen. Finally, in the current study, we were unable to examine lifetime and environmental factors that may provide cognitive reserve, as our sample has high levels and low variability in potential protective factors such as education and socioeconomic status. Future research in samples more accurately reflecting the diversity of the aging population would be better suited to answer the important question of which factors contribute to or detract from cognitive reserve.

In summary, we provide evidence that characteristics of functional brain networks reflecting high levels of efficiency and optimal organization may provide resilience to the accumulation of Aβ pathology, enabling preserved levels of memory. Future research using longitudinal data will better elucidate the temporal relationship between Aβ accumulation and functional network structure to better establish how resilience and/or compensatory mechanisms occur. Graph metrics such as modularity and local efficiency derived from resting state fMRI may be compelling targets for intervention and provide a potentially modifiable link between pathology development and cognitive decline.

## Methods

### Participants

Participants were cognitively normal older adults aged ≥ 60 years from the Biomarker Exploration in Aging, Cognition, and Neurodegeneration (BEACoN: NIA R01AG053555, PI: Yassa) study. Inclusion criteria for BEACoN includes age ≥ 60 years, performance on cognitive assessments within age-adjusted normal range (within 1.5 standard deviations), no major health problems (e.g. uncontrolled diabetes mellitus, uncontrolled hypertension), co-morbid neurological disease (e.g. brain cyst, tumor, aneurysm), or significant psychiatric disorders (e.g. major depressive disorder or attention-deficit hyperactivity disorder), no use of medication for anxiety or depression or illicit drugs, and no MRI or PET contraindications. All participants provided written informed consent. All experimental protocols were approved by the Institutional Review Board (IRB) of the University of California, Irvine, and all methods were carried out in accordance with relevant guidelines and regulations of the IRB.

Seventy-nine participants had both 18F-florbetapir (FBP) PET and high-resolution resting state fMRI and were selected for the present analysis. A total of 14 participants were excluded due to poor resting state fMRI quality (i.e. poor field of view, n = 4; motion; n = 10; see below for details of criteria). Data from 65 participants were included in the final analyses.

### Neuropsychological assessment

Participants received standard neuropsychological assessment, including the Rey Auditory Verbal Learning Test (RAVLT)^56^ and the Mini Mental State Examination^57^. As our primary outcome measure of memory performance, we used RAVLT immediate free recall score after the last learning trial.

### MRI acquisition

Participants received structural and resting state functional MRI at the Campus Center for Neuroimaging (CCNI) at UC Irvine on a 3T Prisma scanner (Siemens Medical System, Munich, Germany) equipped with a 32-channel head coil. A whole brain, high resolution T1-weighetd volumetric magnetization prepared rapid gradient echo images (MPRAGE) was acquired for structural analyses (voxel size = 0.8 mm^3^ resolution, TR/TE/TI = 2300/2.38/902 ms, flip angle = 8°, 240 slices acquired sagittally). High resolution T2*-weighted echo planar images (EPI) were acquired to assess FC (voxel size = 1.8 mm^3^, TR/TE = 2500/26 ms, flip angle = 70°, 39 slices, R >  > L phase encode, partial acquisition covering temporal lobe, 84 volumes). During acquisition, participants were instructed to remain awake and focus on a fixation cross on the screen. High resolution 3D T2-weighted turbo spin echo (TSE) images were acquired in oblique coronal orientation (voxel size = 0.4 × 0.4 mm in-plane resolution, slice thickness = 2 mm, TR/TE = 5000/84 ms, 23 slices) were also acquired to estimate hippocampal volume.

### Structural MRI processing

T1 images were processed with Statistical Parametric Mapping (SPM, version 12, Wellcome Trust Center) and segmented into gray, white, and CSF compartments. T1 images were then warped to MNI152 2 mm standard space using SPM and a study specific DARTEL template. T1 images were also processed with FreeSurfer v.6.0^58^ to obtain a native space regions of interest for FBP quantification.

To obtain a measure of hippocampal volume, T1 and T2 structural images were processed using Automated Segmentation of Hippocampal Subfield (ASHS) software^59^. Volumes of the bilateral DG, CA3, CA2, CA1, and subiculum were added and normalized by the total intracranial volume to estimate hippocampal volume.

### Resting state functional MRI processing

rsfMRI data was preprocessed with SPM12 using a standard pipeline including slice time correction, realignment, and coregistration to the T1 structural image. No spatial smoothing was performed to maintain the high resolution of the images and enable more accurate quantification of signal within spatially adjacent ROIs. Functional images were warped to MNI152 2 mm space using estimates derived from the T1 warping.

Resting state fMRI data were denoised using the CONN toolbox^60^ (version 20) implemented in Matlab version 2019b (The MathWorks, Inc, Natick, MA). Outlier volumes were detected using Artifact Detection Tools (ART) implemented within CONN using conservative threshold of motion > 0.5 mm/TR and a global intensity z-score of 3. Ten subjects were flagged for > 20% volumes detected as outliers and were removed from further analyses^48,50,61^. Denoising included the six realignment parameters and their first-order derivatives (translations and rotations), spike regressors generated from outlier detection^62,63^, anatomical CompCor^64^ (first five components of time series signal from white matter and CSF), bandpass filtering [0.008–0.1 Hz], and linear detrending applied to the residual time series.

To determine ROI inclusion, we first created an explicit mask of common coverage across the partial field of view of the rsfMRI scan. Four participants were excluded prior to mask creation due to their field of view not including critical medial temporal lobe structures such as the hippocampus. We next applied this explicit mask to the Brainnetome Atlas^65^ to determine which ROIs had sufficient coverage. We excluded any ROIs with < 50% retention within the bounds of our explicit mask, resulting in 135 ROIs that were sufficiently included within the field of view for all participants (see Supplementary Table 1 for a list of ROIs included, and Fig. 1A for a visualization).

BOLD time series were extracted after denoising from the 135 Brainnetome ROIs, and used to perform ROI-to-ROI first-level analyses in CONN. This resulted in a 135 × 135 functional connectivity matrix for each participant, which reflected Fisher’s r-to-z transformed correlation coefficients for each ROI pair.

### Graph analyses

The Brain Connectivity Toolbox (BCT), implemented in Matlab 2019b, was used for graph analyses. ROI-to-ROI functional connectivity matrices were first optimized for analyses by symmetrizing the matrix and ensuring the diagonals were set to zero. Adjacency matrices were created by binarizing across costs ranging from 5% of the top connections in the network (most sparse) to the top 35% of the network (less sparse) in step sizes of 5%, resulting in seven total costs (see Fig. 1B). This range was selected due to small-world network characteristics occurring at low-to-medium cost levels, up to approximately 30–35% sparsity^19,24,33^, and values stabilizing around this level (see Fig. 1D). We conducted analyses across seven total costs to provide statistical evidence that our results are robust to an arbitrary cost selection, indicated by the lack of a significant main effect of cost in the repeated measures ANCOVA analyses. We focused on three main graph theoretical measures: local efficiency, modularity, and small worldness.

#### Local efficiency

Local efficiency was calculated with the “efficiency_bin” BCT function, resulting in one value per ROI. For primary analyses, local efficiency was averaged across all ROIs to get one value representing the network. We also averaged local efficiency over Brainnetome ROIs included in the default mode network (DMN) and visual network separately as defined by Yeo and colleagues^66^. Finally, we averaged local efficiency over the four Brainnetome ROIs representing the hippocampus (rostral and caudal hippocampal ROIs, left and right) for a whole-hippocampal measure of local efficiency, as well as averaging across bilateral superior temporal gyrus ROIs (A22c left and right) as a control region.

#### Modularity

Modularity was calculated with the “community_louvain” BCT function. Parameters included the default “modularity” setting, with a gamma value of 1.0. The modularity variable “Q” was estimated for the full network of included ROIs and used in analyses as the outcome measure.

#### Small worldness

Small worldness is calculated by normalizing the clustering coefficient and path length of the observed network to a random network, and then taking the ratio of the normalized clustering coefficient to the normalized path length^21,26^. First, to calculate the observed clustering coefficient (C) and path length (L), we calculated the average C and L of all ROIs in the network for each participant at each cost with the BCT. Second, we constructed a random network that had the same number of edges as our observed networks but with all connections rewired using the “randomizer_bin_und” BCT function. We then calculated the average clustering coefficient and path length for this random network. This process was iterated 10,000 times to generate a distribution of null clustering coefficient and path length values, and the average null clustering coefficient (C_0_) and path length (L_0_) was calculated at each cost. Finally, for each participant and cost, we calculated small worldness of the network with the following formula: σ = (C/C_0_)/(L/L_0_).

### Aβ PET

Participants received 18F-florbetapir PET (FBP) at the CCNI with an ECAT High Resolution Research Tomograph (HRRT, CTI/Siemens, Knoxville, TN, USA). Ten mCi of tracer was injected, and four five-minute frames were collected between 50 and 70 min post-injection. FBP data was reconstructed with attenuation correction, scatter correction, and 2 mm^3^ Gaussian smoothing. Frames were realigned, averaged, coregistered to the T1 MRI, and normalized by a whole cerebellum reference region to compute SUVR images. Additional 6 mm^3^ smoothing was then applied to achieve an effective resolution of 8 mm^3^. We calculated a global measure of FBP SUVR across a previously validated cortical composite region^32^. Aβ+ status was determined using a threshold of > 1.11 global FBP SUVR^32^.

### Statistical analysis

Statistical analyses were performed using jamovi v1.6 (https://www.jamovi.org) and RStudio v1.4. Demographics and memory performance between Aβ groups were compared with independent samples t-tests. Repeated-measures ANCOVA models were constructed to test associations for each graph metric, separately. The graph metric at each cost (7 costs) was included as a repeated within-subjects factor, Aβ status was included as a between-subjects factor, memory or hippocampal volume as the variable of interest, and age, sex, and education as covariates of no interest. In cases where the assumption of sphericity was violated (Maulchy’s *W p* < 0.05), Greenhouse-Geisser corrections were applied. Main effects and interactions were considered significant at *p* < 0.05.

When interactions crossed this significance threshold, we conducted planned follow-up analyses to further isolate the effects. In the case of interactions involving Aβ status, we conducted follow-up interactions using linear regression models at each cost to test the specificity of the effect, including the interaction of interest and age, sex, and education as covariates. To further test which group was driving each interaction, we conducted partial correlations covarying for age, sex, and education within each Aβ group separately. To test if the correlation between memory and each graph metric was significantly different in each Aβ group, correlation strengths (Fisher’s r to z transformation) were compared with the “cocor” package^67^ in RStudio.

### Supplementary Information

Supplementary Tables.

## Data Availability

The datasets generated during and/or analyzed during the current study are available from the corresponding author on reasonable request.
