# Supplementary material for: Functional network structure supports resilience to memory deficits in cognitively normal older adults with amyloid-β pathology
Source: Sci Rep. 2023 Aug 25;13:13953. doi: 10.1038/s41598-023-40092-x (PMC10457346; doi:10.1038/s41598-023-40092-x)
Supplement: Supplementary file 1 — Supplementary Tables. [file 41598_2023_40092_MOESM1_ESM.pdf]

Supplementary Table 1. Regions of interest (ROI) from the Brainnetome Atlas included in the group explicit mask for functional analyses.

Region	Total ROIs	Included ROIs	% Included
Frontal Lobe	68	24	35.29%
Superior Frontal Gyrus	14	0	0.00%
Middle Frontal Gyrus	14	2	14.29%
Inferior Frontal Gyrus	12	9	75.00%
Orbital Gyrus	12	11	91.67%
Precentral Gyrus	12	2	16.67%
Paracentral Lobule	4	0	0.00%
Temporal Lobe	56	43	76.79%
Superior Temporal Gyrus	12	11	91.67%
Middle Temporal Gyrus	8	8	100.00%
Inferior Temporal Gyrus	14	7	50.00%
Fusiform Gyrus	6	4	66.67%
Parahippocampal Gyrus	12	9	75.00%
Posterior Superior Temporal Sulcus	4	4	100.00%
Parietal Lobe	38	2	5.26%
Superior Parietal Lobe	10	0	0.00%
Inferior Parietal Lobe	12	0	0.00%
Precuneus	8	0	0.00%
Postcentral Gyrus	8	2	25.00%
Cingulate Gyrus	26	17	65.38%
Insular Gyrus	12	12	100.00%
Cingulate Gyrus	14	5	35.71%
Occipital Lobe	22	13	59.09%
Medioventral Occipital Cortex	10	9	90.00%
Lateral Occipital Cortex	12	4	33.33%
Subcortical Regions	36	36	100.00%
Amygdala	4	4	100.00%
Hippocampus	4	4	100.00%
Basal Ganglia	12	12	100.00%
Thalamus	16	16	100.00%
Total	246	135	54.88%

Supplementary Table 2. Repeated measures ANCOVA results predicting local efficiency in control networks/regions.

Network/Region	Model Term	F	df	p
Visual Network	Aβ status[a]	1.60	1	0.21
	Aβ status x Cost[a]	0.43	1.44	0.59
	Memory[b]	0.59	1	0.44
	Memory x Cost[b]	0.85	1.43	0.40
	Memory x Aβ status[c]	2.85	1	0.10
	Memory x Aβ status x Cost[c]	0.62	1.40	0.49
Superior Temporal Gyrus	Aβ status[a]	0.03	1	0.86
	Aβ status x Cost[a]	0.25	1.39	0.69
	Memory[b]	6.41	1	0.01
	Memory x Cost[b]	1.33	1.38	0.26
	Memory x Aβ status[c]	0.10	1	0.76
	Memory x Aβ status x Cost[c]	0.12	1.37	0.81

The visual network was chosen as a control for the default mode network, and the superior temporal gyrus was chosen as a control for the hippocampus.

[a]Repeated measures ANCOVA models included Aβ status, age, sex, and education, predicting to local efficiency across seven costs as the repeated measures factor.

[b]Repeated measures ANCOVA models included memory, age, sex, and education, predicting to local efficiency across seven costs as the repeated measures factor.

[c]Repeated measures ANCOVA models included memory x Aβ status interaction, age, sex, and education, predicting to local efficiency across seven costs as the repeated measures factor.

Supplementary Table 3. Repeated measures ANCOVA results for hippocampal volume.

Graph Metric	Model Term	F	df	p
Local Efficiency	Hippocampal Volume[a]	0.27	1	0.61
	Hippocampal Volume x Cost[a]	0.41	1.84	0.65
	Memory x Hippocampal Volume[b]	0.88	1	0.35
	Memory x Hippocampal Volume x Cost[b]	0.57	1.83	0.55
Modularity	Hippocampal Volume[a]	0.02	1	0.88
	Hippocampal Volume x Cost[a]	0.36	1.41	0.63
	Memory x Hippocampal Volume[b]	1.14	1	0.29
	Memory x Hippocampal Volume x Cost[b]	0.21	1.39	0.73
Small Worldness	Hippocampal Volume[a]	1.15	1	0.29
	Hippocampal Volume x Cost[a]	0.17	1.49	0.78
	Memory x Hippocampal Volume[b]	1.06	1	0.31
	Memory x Hippocampal Volume x Cost[b]	0.23	1.49	0.73
Local Efficiency (Hippocampus)	Hippocampal Volume[a]	0.01	1	0.93
	Hippocampal Volume x Cost[a]	0.06	1.63	0.91
	Memory x Hippocampal Volume[b]	0.02	1	0.89
	Memory x Hippocampal Volume x Cost[b]	0.20	1.62	0.77

[a]Repeated measures ANCOVA models included hippocampal volume, age, sex, and education, predicting to each graph metric across seven costs as the repeated measures factor.

[b]Repeated measures ANCOVA models included hippocampal volume x memory interaction, age, sex, and education, predicting to each graph metric across seven costs as the repeated measures factor.
